# Supplementary material for: The interactome of intact mitochondria by cross-linking mass spectrometry provides evidence for coexisting respiratory supercomplexes
Source: Mol Cell Proteomics. 2017 Dec 8;17(2):216–32. doi: 10.1074/mcp.RA117.000470 (PMC5795388; doi:10.1074/mcp.RA117.000470)
Supplement: Supplemental Data [file supp_RA117.000470_133922_0_supp_23978_fzffwf.zip › spectra_annotation/mito_spectra_annotation/37-1-14-1-3-1.pdf]

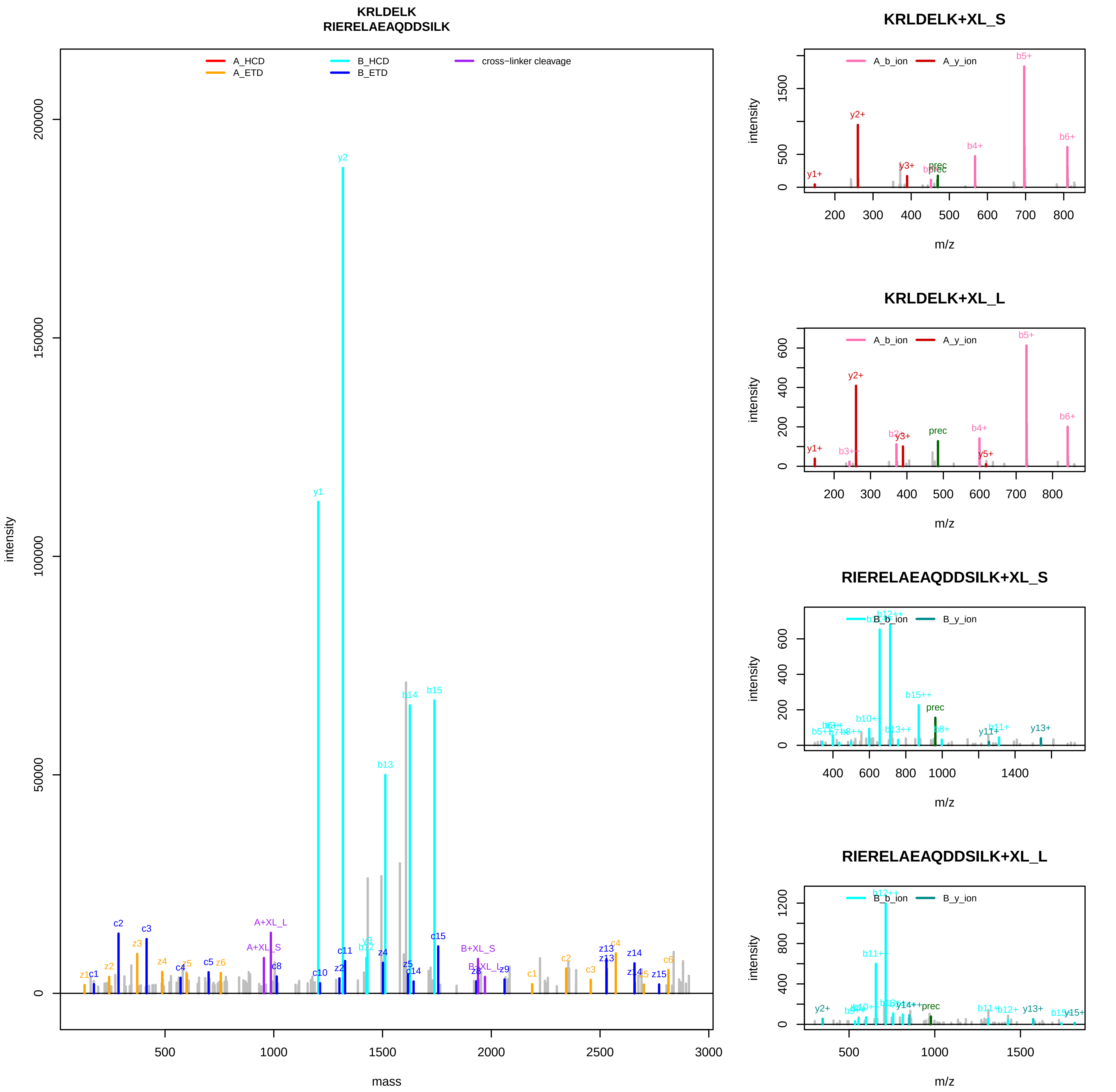

KRLDELK
RIERELAEAQDDSILK
A_HCD
A_ETD
B_HCD
B_ETD
cross−linker cleavage
intensity
mass
0
50000
100000
150000
200000
500
1000
1500
2000
2500
3000
y2
y1
b13
b14
b15
A+XL_L
A+XL_S
B+XL_S
c2
c3
c11
c15
z13
z14
KRLDELK+XL_S
A_b_ion
A_y_ion
b5+
b6+
b4+
y2+
y3+
y1+
prec
m/z
KRLDELK+XL_L
y5+
RIERELAEAQDDSILK+XL_S
B_b_ion
B_y_ion
b12++
b15++
b10++
b13++
b11+
y11+
y13+
RIERELAEAQDDSILK+XL_L
b11++
y14++
b12+
y2+
